# Affect dysregulation, psychoform dissociation, and adult relational fears mediate the relationship between childhood trauma and complex posttraumatic stress disorder independent of the symptoms of borderline personality disorder

**DOI:** 10.1080/20008198.2017.1400878

**Published:** 2018-01-23

**Authors:** Annemiek van Dijke, Juliette A. B. Hopman, Julian D. Ford

**Affiliations:** ^a^ Yulius Academy/Yulius/COLK Centre for Psychosomatics Yulius, Dordrecht - Zaandam, the Netherlands; ^b^ Department of Clinical Psychology, VU University Amsterdam, Rotterdam, the Netherlands; ^c^ PsyQ Zaandam & Parnassia Academy, the Netherlands; ^d^ Department of Medical Psychology and Psychotherapy, Erasmus MC, Rotterdam, the Netherlands; ^e^ Department of Psychiatry, University of Connecticut Health Center, Farmington, CT, USA

**Keywords:** Complex posttraumatic stress disorder, PTSD, childhood trauma, BPD, dissociation, attachment, affect dysregulation, alexithymia, emotion regulation, somatization, trastorno de estrés postraumático complejo, TEPT-C, DESNOS, TEPT, trauma infantil, TLP disociación, apego, desregulación afectiva, alexitimia, regulación de las emociones, somatización., 复杂创伤后应激障碍, CPTSD, DESNOS, PTSD, 童年创伤, BPD, 分离, 依恋, 情感失调, 抒情障碍, 情绪调控, 躯体化, • Assessing the relations between childhood trauma and adult complex cPTSD direct and indirect through mediators affect dysregulation, dissociation and attachment fears., • Assessing these direct and indirect relations while correcting for BPD-symptoms., • Assessing similarities and differences for the direct and indirect relations between childhood trauma and cPTSD and/or BPD.

## Abstract

**Objective**: Complex posttraumatic stress disorder (CPTSD) as defined by the Disorders of Extreme Stress Not Otherwise Specified (DESNOS) formulation is associated with childhood relational trauma and involves relational impairment, affect dysregulation, and identity alterations. However, the distinct contributions of relational impairment (operationalized in the form fears of closeness or abandonment), affect dysregulation (operationalized in the form of overregulation and under-regulation of affect), and identity alterations (operationalized in the form of positive or negative psychoform or somatoform dissociation) to the relationship between childhood trauma and CPTSD/DESNOS have not been systematically tested.

**Method and Results**: In a clinical sample of adults diagnosed with severe and chronic psychiatric and personality disorders (*n* = 472; *M* = 34.7 years, *SD* = 10.1), structural equation modelling with bootstrap 95% confidence intervals demonstrated that the association between childhood trauma and CPTSD/DESNOS symptoms in adulthood was partially mediated by under-regulation of affect, negative psychoform dissociation, and adult relational fears of closeness and of abandonment. These results also were independent of the effects of borderline personality disorder (BPD) symptoms.

**Conclusions**: Some, but not all, hypothesized components of the DESNOS formulation of CPTSD statistically mediate the relationship between childhood trauma and adult CPTSD/DESNOS. These relationships appear specific to CPTSD/DESNOS and not to the effects of another potential sequelae of childhood trauma BPD. Replication with prospective longitudinal studies is needed.

## Abstract (es)

**Planteamiento**: El trastorno por estrés postraumático (TEPT complejo), tal como se define en la formulación trastornos por estrés extremo no especificado (DESNOS) se asocia con trauma relacional infantil e implica un deterioro relacional, desregulación afectiva y alteraciones de identidad. Sin embargo, las distintas contribuciones del deterioro relacional (operacionalizado en la forma de temor a la cercanía o al abandono), la desregulación afectiva (operacionalizada en forma de sobrerregulación y subregulación afectiva) y las alteraciones de identidad (operacionalizadas en forma de disociación psicomorfa o somatoforme positiva o negativa) a la relación entre trauma infantil y TEPT-C/DESNOS no han sido probadas sistemáticamente.

**Método y Resultados**: En una muestra clínica de adultos diagnosticados con trastornos psiquiátricos y de personalidad graves y crónicos (*n* = 472; *M* = 34,7 años, DE = 10,1), el modelo de ecuaciones estructurales con bootstrap del 95% - intervalos de confianza demostraron que la asociación entre el trauma infantil y los síntomas de TEPT-C/DESNOS en la edad adulta estaba parcialmente mediada por una regulación afectiva insuficiente, la disociación psicomorfa negativa y temores relacionales adultos de cercanía y abandono). Estos resultados también fueron independientes de los efectos de los síntomas del trastorno del límite de la personalidad (TLP).

**Conclusiones**: Algunos, pero no todos, plantearon la hipótesis co de la formulación del DESNOS del TEPT-C median estadísticamente en la relación entre trauma infantil y el TEPT-C/DESNOS adulto. Estas relaciones parecen ser específicas de TEPT-C/DESNOS y no de los efectos de otras posibles secuelas del trauma infantil, el TLP. Es necesario replicarlo con estudios longitudinales prospectivos.

## Abstract (zh)

**背景**：复杂创伤后应激障碍(CPTSD)定义为：未另做说明的极端应激引起的失调(DESNOS)。它和童年关系创伤相关，包括了人际损伤、情感失调和同一性改变。但是，人际损伤(害怕亲密或被抛弃)、情感失调(过度调节和调节不足)和同一性改变(正性和负性的心理形式或者躯体形式分离)分别在童年创伤和CPTSD/DESNOS关系中的作用还没有被系统研究过。

**方法和结果**：在一个临床成人样本，诊断为严重和长期的精神病和人格障碍(*n* = 472; *M* = 34.7 years, *SD* = 10.1)。使用95%置信区间bootstrap结构方程模型结果表明，童年创伤和CPTSD/DESNOS 症状的关联被情感调节不足、负性心理形式分离和成年期对亲密和抛弃的关系恐惧部分中介。这些结果独立于边缘性人格障碍(BPD)症状。

**结论**：CPTSD 的DESNOS表述的一些(并非全部)假设成分中介童年创伤和成年CPTSD/DESNOS的关系。只有CPTSD/DESNOS和童年创伤关联，而童年创伤的另一个可能后果BPD 没有。需要在回溯追踪研究中进行重复验证。

The lifespan sequelae of exposure to interpersonal psychological trauma (emotional or physical neglect or abuse, or sexual abuse) in childhood include not only posttraumatic stress disorder (PTSD) but also a plethora of symptoms and mental disorders that may occur comorbidly with or separately from PTSD (D’Andrea, Ford, Stolbach, & van der Kolk, 2012). Whether these sequelae are best understood as a complex variant of PTSD (CPTSD) or a complicated array of overlapping psychiatric and personality disorders is controversial (Landy, Wagner, Brown-Bowers, & Monson, 2015; Resick et al., 2012). However, there is mounting evidence that a Disorders of Extreme Stress Not Otherwise Specified (DESNOS) formulation of CPTSD constitutes a distinct syndrome of potential clinical utility (e.g. Ford & Courtois, 2014; Herman, 2012).

Three core features of the DESNOS formulation of CPTSD symptomatology were identified based on a comprehensive literature review (Ford, 2017a): affect dysregulation, identity alterations, and relational impairment (Ford, 2015; Knefel, Garvert, Cloitre, & Lueger-Schuster, 2015). Affect dysregulation is defined as problems in managing or recovering from extreme states of affect, including both under-regulation of heightened affect states and maladaptive overregulation of affect (Pat-Horenczyk et al., 2015; Van Dijke et al., 2010). Under-regulation involves limited access to or capacity for deploying strategies to reduce intense affect states and associated difficulties with impulse control and goal-directed behaviour (e.g. anger that escalates into rage, or anxiety that becomes an unmanageable state of terror). Overregulation involves non-acceptance and limited awareness or clarity of emotions (e.g. states of profound emotional emptiness or detachment). Identity alterations involve problems with maintaining a coherent sense of self, which may take the form of dissociation symptoms including somatoform or embodied dissociative symptoms such as conversion symptoms, and psychoform or mentalized dissociative symptoms such as depersonalization, amnesia or identity alterations that may turn into positive or negative forms of dissociation (e.g. Van Dijke, Van Dijke, Ford, Frank, Van der Hart, 2015; Van Dijke et al., 2010). Relational impairment in adulthood involves two dimensions (Van Dijke & Ford, 2015) that have been shown to have better internal consistency than the prototypical secure, preoccupied, dismissing, and unresolved attachment categories and to provide a good fit in confirmatory factor analyses: avoidance (i.e. fear of closeness) and anxiety (i.e. fear of abandonment) (Roisman et al., 2007). Attachment-related avoidance and anxiety were selected to represent adult relational impairment rather than the childhood-based categories of insecure attachment because they were shown to be trait-like risk factors for self-reported psychiatric symptoms (i.e. correlated with psychopathology under conditions of both high and low stress), while the insecure attachment categories were associated with psychopathology in adults only under high stress conditions (Fortuna & Roisman, 2008).

The DESNOS formulation of CPTSD in adulthood has been demonstrated empirically to be associated with childhood relational experiences that are potentially traumatic (e.g. maltreatment, family violence) consistently across numerous studies (Cloitre, Garvert, Brewin, Bryant, & Maercker, 2013; Cloitre, Garvert, Weiss, Carlson, & Bryant, 2014; Dorahy et al., 2014; Knefel et al., 2015; Knefel & Lueger-Schuster, 2013; Wolf et al., 2015). CPTSD as defined by DESNOS is theorized to represent the results of developmental adaptations to exposure to interpersonal trauma in developmentally sensitive periods, including altered emotion processing (Langevin, Hebert, & Cossette, 2015; Shields & Cicchetti, 1998), dissociative shifts in self-awareness and consciousness (Bailey, Moran, & Pederson, 2007; Lyons-Ruth, Dutra, Schuder, & Bianchi, 2006), and disruption of secure attachment working models (Madigan, Vaillancourt, McKibbon, & Benoit, 2012; Stronach et al., 2011). However, whether there is a specific linkage between exposure to potentially traumatic events in childhood and each of the proposed core features of CPTSD as defined by DESNOS has not been systematically investigated.

In addition, borderline personality disorder (BPD) arguably involves similar forms of dysregulation (e.g. Cloitre et al., 2014; Van Dijke et al., 2012) and, therefore, whether the DESNOS formulation of CPTSD-symptoms can be considered distinct from BPD symptoms warrants careful research (Ford & Courtois, 2014; Van Dijke et al., 2012). Interpersonal victimization in childhood is highly prevalent among (young) adults with BPD and adolescents with BPD symptoms. Adults with BPD are more likely than those with other mental disorders or non-clinical controls to report childhood emotional abuse and neglect or impaired caregivers, leading to the hypothesis that traumatic victimization and compromised primary caregiving relationship are aetiological contributors to BPD (e.g. Van Dijke et al., 2011). Although severe childhood sexual abuse (i.e. prolonged, violent, multiple perpetrators, physical penetration) are the childhood trauma type most consistently associated with BPD symptoms and impairment, neglect (which often co-occurs with sexual abuse) is a separate risk factor for BPD (e.g. Van Dijke et al., 2012). Nevertheless, BPD and CPTSD can be distinguished in terms of clinical phenomenology. CPTSD as defined by DESNOS appears to involve hypervigilance related to being harmed, whereas BPD involves extreme sensitivity to perceiving oneself as being abandoned or rejected/shamed (Ford & Courtois, 2014; Van Dijke et al., 2012).

The present study was conducted to empirically determine whether these three forms of dysregulation characteristic of both BPD and the DESNOS formulation of CPTSD mediate the relationship between exposure to interpersonal traumatic stressors in childhood and CPTSD symptoms independent of the effects of BPD symptoms, with a clinical sample of adults diagnosed with severe and chronic psychiatric and personality disorders. Based on clinical and phenomenological differences between the DESNOS formulation of CPTSD and BPD, we hypothesized that: (1) overregulation and under-regulation of affect would be a mediator of the relationship between childhood trauma and CPTSD, because the DESNOS formulation of CPTSD (but not BPD) includes features of emotional numbing that are consistent with over-regulated affect; (2) positive and negative somatoform dissociation would be a mediator, because the DESNOS formulation of CPTSD includes symptoms of somatic dysregulation that are not included in BPD; (3) for CPTSD, fear of closeness, but not of abandonment, would be a mediator, because the DESNOS formulation of CPTSD includes symptoms of interpersonal detachment while BPD is characterized by fear of abandonment and rejection (Ford & Courtois, 2014).

## 1. Method

### 1.1. Procedure and participants

Adult psychiatric patients participated in this naturalistic inpatient psychotherapy project in a tertiary mental health institution following a protocol approved by the Dutch medical ethics committee for mental health research (METiGG). In line with the Declaration of Helsinki, all participants provided written informed consent to participate after the procedure had been fully explained. DSM-IV-TR diagnoses were assessed at intake by psychiatrists and psychotherapists. Next, structured clinical interviews were conducted by trained interviewers supervised by the first author, during an initial diagnostic and evaluation phase following the inpatient psychiatric admission. For inclusion, all participants met DSM-IV criteria for BPD or generalized anxiety or unipolar depressive disorders, with sufficient psychosocial impairment and chronicity to require multiple previous episodes of inpatient and outpatient psychiatric care and referral for specialized tertiary clinical psychotherapy.

Exclusion criteria were assessed, including: history of potential brain damage (e.g. head injury, electroshock therapy), psychotropic medications that may impair executive function (e.g. anti-psychotics, lithium), severe mental illness (i.e. severe dissociative disorder, bipolar disorder, schizophrenia, other psychotic disorders), eating disorder with severe underweight, imminent suicidality, and developmental disorders (i.e. autism spectrum disorders or attention-deficit hyperactivity disorder).

Demographic and diagnostic characteristics are presented in Table 1. Age and sex were significantly related to several primary study variables (i.e. age with CPTSD symptoms, *r* = -.16, fear of abandonment, *r* = -.28, fear of closeness, *r* = -.11, affect instability, *r* = -.19, negative psychoform dissociation, *r* = -.16, and positive psychoform dissociation, *p*s ranging from < .001 to .026); sex with CPTSD symptoms, *r* = .13, fear of closeness, *r* = .18, positive psychoform dissociation, *r* = .12, and positive somatoform dissociation, *r* = 10, *p*s ranging from < .001 to .031). These characteristics were used as covariates in subsequent analyses. As no significant relationships were found between the study variables and marital status or education level, these variables were not used as covariates.

**Table 1.** Demographic and diagnostic differences between adult patients with or without CPTSD (*n* = 449).

		Presence of CPTSD						
		yes (*n* = 126)		no (*n* = 323)		Test of differences		
		*M*	*SD*	*M*	*SD*	*F*	*df*	*p*
Age		32.9	9.9	35.2	10.2	4.83	447	.03
		*n*	*%*	*n*	*%*	χ^2^	*df*	*p*
Sex	Male	32	25	107	33	2.53	1	.11
Marital	NP	63	50	160	50	.49	3	.69
status	LT	15	12	33	10			
	SDD	16	13	35	11			
	Married	32	25	95	29			
Educational	Low	36	29	74	23	3.92	2	.14
level	Middle	42	33	140	43			
	High	48	38	109	34			
Diagnoses	BPD	41	33	75	23	45.70	3	<.001
	BPD + SoD	58	46	70	22			
	SoD	17	14	136	42			
	A/AD	10	8	42	13			

NP = no primary partner, LT = living together, SDD = separated by death or divorce, Low = primary and low-level secondary education, Middle = middle-level secondary education, High = high-level secondary education, BPD = borderline personality disorder, SoD = primary somatoform disorder, A/AD = primary affective or anxiety disorder; CPTSD = complex posttraumatic stress disorder.

### 1.2. Measures

*C* *PTSD-symptoms* as defined by DESNOS were confirmed using the Dutch self-report version of the Structured Interview for Disorders of Extreme Stress Not Otherwise Specified (SIDES-rev; Ford & Kidd, 1998; Van Dijke & Van der Hart, 2002). The SIDES-rev-NL total score was internally consistent in this sample (Cronbach’s alpha = 0.91). Current rather than lifetime CPTSD was assessed to reduce reliance on retrospective reports. Partial (Ford & Smith, 2008) as well as full CPTSD was included, requiring the presence of at least two of the primary CPTSD/DESNOS features criteria (i.e. affect dysregulation, dissociation, somatization) and one of the three altered fundamental beliefs subscales (i.e. self, relationships, systems of meaning). Symptoms were confirmed by clinical observation and multi-disciplinary consensus during inpatient pretreatment phase (van Dijke, 2008).

*BPD symptoms* were confirmed using the Personality Disorder Severity Index (BPDSI; Weaver & Clum, 1993; Dutch version IV, Arntz et al., 2003). The BPDSI is a semi-structured interview that contains nine sections (abandonment, relationships, self-image, impulsivity, parasuicide, affect, emptiness, anger, dissociation and paranoia) corresponding to the symptom clusters of BPD. The BPDSI has good validity and reliability (Arntz et al., 2003). Cronbach’s alphas were adequate (for the subscales ranging from .70 to .93; and for the total score .96).

*Childhood trauma* was measured using the Traumatic Experiences Checklist (TEC; Dutch version, Nijenhuis, Van der Hart, & Kruger, 2002), a retrospective self-report questionnaire concerning adverse experiences and potential traumatic events. Reports of potential traumatic events were confirmed by close relatives in a sub-sample of *n *= 354 participants. A total childhood trauma exposure score was calculated by the sum of all scores, adding all positive items. The TEC has demonstrated reliability and validity with psychiatric outpatients (Nijenhuis et al., 2002).

The mediators were assessed in their separate inhibitory and excitatory forms:

*Problems in affect regulation involve under-regulation and over-regulation of affect* (*van Dijke, van der Hart, et al.*, 2010 *).* Over-regulation of affect was in all participants assessed with the Bermond Vorst Alexithymia Questionnaire (BVAQ; Vorst & Bermond, 2001), a Dutch forty-item questionnaire with demonstrated psychometric qualities (Vorst & Bermond, 2001). The Cognitive (inhibited verbalizing, identifying, and analysing emotions) over-regulation sub-scale was used to enable comparisons with previous studies (Waller & Scheidt, 2004, 2006) and based on its strong correlation with the Toronto Alexithymia Scale (TAS-20; Bagby, Parker, & Taylor, 1994; r = .80). The BVAQ cognitive scale had good internal consistency reliability in the present sample (Cronbach’s alpha = .88). Under-regulation of affect in BPD-participants was assessed with the ‘Dysregulated affect sub-scale’ from the SIDES-rev-NL, which is internally consistent (Cronbach’s alpha = .75) and has shown evidence of convergent and discriminant validity (Van Dijke, Van der Hart, et al., 2010) and construct validity (Van Dijke et al., 2011, Van Dijke et al., 2012) with the current study sample. Under-regulation in CPTSD-patients was assessed with the ‘Affect instability scale’ from the BPDSI. BPDSI scores range from 0 = never to 10 = daily (Cronbach’s alpha = .81).

*Relational impairment*. Adult relational fears involve ‘fear of abandonment’ and ‘fear of closeness’ (e.g. Van Dijke & Ford, 2015), which were assessed using the Dutch version of the validated 30-item Relationship Style Questionnaire (RSQ; Griffin & Bartholomew, 1994; Van Dijke, 2002). Dimensional scores (Fortuna & Roisman, 2008) were calculated for fear of abandonment (attachment-related anxiety; Cronbach’s alpha = .74) and fear of closeness (attachment-related avoidance; Cronbach’s alpha = .72).

*Dissociation* involves positive and negative psychoform and somatoform features (Van Dijke et al., 2010). Dissociation was assessed with the Dissociative Experiences Scale (DES; Bernstein & Putnam, 1986; Dutch version, Ensink & Van Otterloo, 1989) for negative (e.g. amnesia) and positive (e.g. intrusions) psychoform features, and with the Somatoform Dissociation Questionnaire (SDQ-20; Dutch version, Nijenhuis et al., 2002) for negative (e.g. anaesthesia, paralysis) and positive (e.g. pain, cramps; Van der Hart, Nijenhuis, & Steele, 2005) somatoform features. The 28-item DES is internally consistent (Cronbach’s alpha in this sample = .95), temporally reliable, and extensively validated (Ensink & Van Otterloo, 1989). The 20-item SDQ-20 is internally consistent (Cronbach’s alpha in this sample = .96) and has demonstrated evidence of construct validity (Nijenhuis et al., 2002).

### 1.3. Data analysis

Using Mplus, version 6.12 (Muthén & Muthén, 1998-2011), mediation analyses were conducted on continuous scores with path analysis. The indirect effects were tested with bootstrap 95% confidence intervals with the bootstrap sample set to 5000 estimates. Indirect effects were considered insignificant when zero appeared in the confidence interval. When conducting the mediation analyses, a statistical approach was used as proposed by Muthén (2011) and Preacher & Hayes (2004). To examine whether assumptions were met to proceed to testing for mediation via problems in relational fears, affect regulation, and dissociation, Pearson’s correlations were used to test if candidate mediators were significantly related to the total trauma score and the total severity score for symptoms of CPTSD/DESNOS. A series of regression analyses were used to test if the candidate mediators that were significantly associated with both the total trauma and CPTSD/DESNOS severity scores partially or fully mediated the relationship between childhood trauma and symptoms of CPTSD/DESNOS, both on an unadjusted basis and when including BPD symptom severity as a separate outcome along with CPTSD/DESNOS symptom severity.

First, a model was specified in which symptoms of CPTSD/DESNOS were predicted by reported childhood trauma directly, controlling for age and sex. Candidate mediators that were significantly correlated to reports of childhood trauma and symptoms of CPTSD/DESNOS were then added to produce the model depicted in Figure 1 Panel 1a. Next, the mediation model was adjusted to account for effects of BPD symptom severity, as depicted in Figure 1 Panel 1b. Finally, to test whether path estimates of the indirect paths between childhood trauma and CPTSD/DESNOS differed between the two mediation models, unstandardized path estimates in the model with BPD and CPTSD/DESNOS were fixed to be equal to the path estimates of the model with only CPTSD/DESNOS, as shown in Figure 1 Panel 1c. Path estimates derived from the models in Figure 1 Panels 1b and 1c were compared with chi-squared tests. A significant chi-squared would indicate that accounting for BPD symptom severity yields different path estimates than when only CPTSD/DESNOS is accounted for. All statistically non-significant associations (*p* > .10) were removed so that only statistically significant paths are depicted in Figure 1.

**Figure 1.**
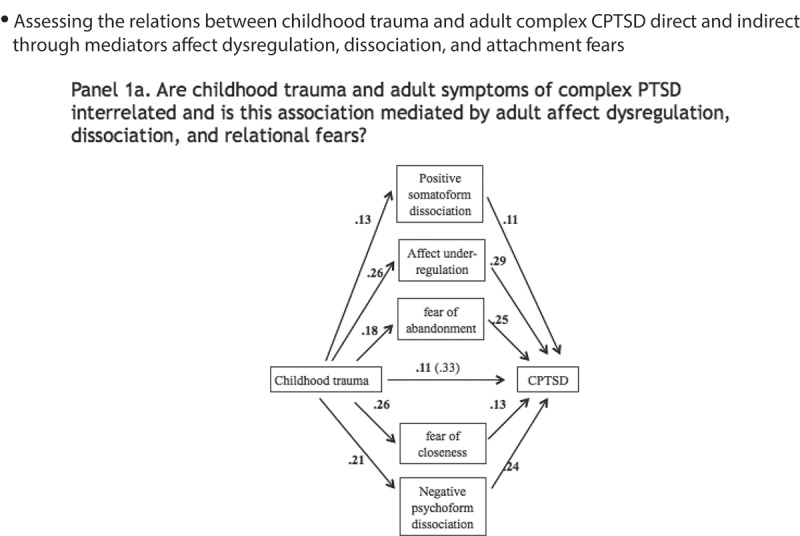
Mediation models. Panel 1a shows the mediation model for the relationship between childhood trauma and adult symptoms of CPTSD via adult affect dysregulation, dissociation, and relational fears. All direct path estimates are depicted as standardized regression weights.Panel 1b shows the mediation model adjusted for BPD symptoms. All direct path estimates are depicted as standardized regression weights. Mediators of relationships between childhood trauma and CPTSD are shown in **bold black** font. Mediators of relationships between childhood trauma and BPD are in grey font. Shared mediators are in *italic*.Panel 1c shows the mediation model adjusted for BPD symptoms with path estimates constrained (@) at values of the unadjusted model. All path estimates are depicted as standardized regression weights. Mediators of relationships between childhood trauma and CPTSD are shown in **bold black** font. Mediators of relationships between childhood trauma and BPD are in grey font. Shared mediators are in *italic.*

**Figure:**
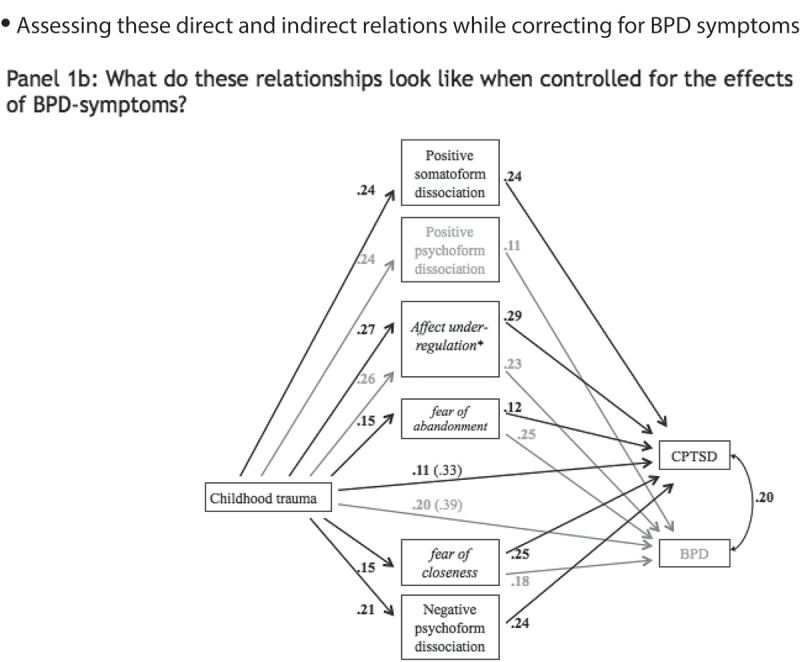

**Figure 1.**
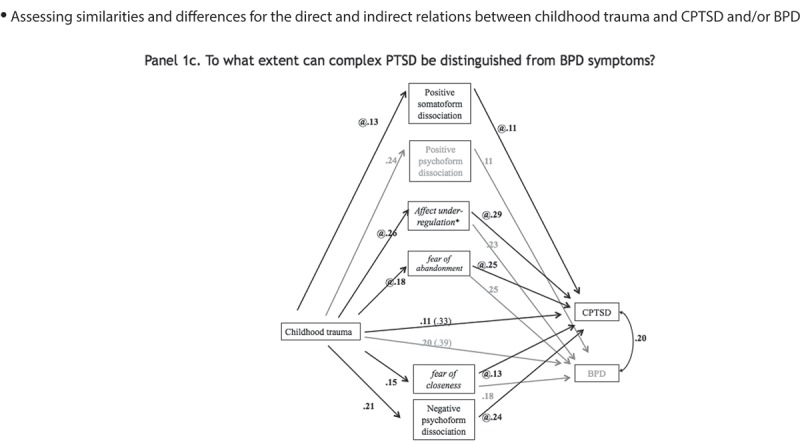
(Continued).

## 2. Results

### 2.1. Descriptive statistics and bivariate correlations

Means and standard deviations of study variables are depicted in Table 2. Support was found to test for mediation between childhood trauma and CPTSD/DESNOS through fear of abandonment and closeness, affect instability, and positive and negative symptoms of psychoform and somatoform dissociation: (1) CPTSD/DESNOS symptoms were positively related to severity of childhood trauma, as well as affect dysregulation, relational fears, and dissociation symptoms; (2) BPD symptoms were positively related to severity of childhood trauma, as well as affect dysregulation, relational fears, and dissociation symptoms; (3) childhood trauma severity was also related to dissociation symptoms, relational fears, and under-regulation of affect, but not overregulation of affect.

**Table 2.** Descriptive statistics and Pearson correlations coefficients of symptoms of complex posttraumatic stress disorder in adulthood, childhood trauma events, and relational fears, affect regulation and dissociation (*n* = 449).

	*M*	*SD*	1	2	3	4	5	6	7	8	9
1. CPTSD symptoms	61.52	15.32									
2. Childhood trauma	7.36	4.92	.**32**								
3. Affect under-regulation	5.26	2.97	.**52**	.**26**							
4. Affect overregulation	75.49	18.20	.**33**	.03	.08						
5. Fear of abandonment	1.89	.75	.**33**	.**25**	.**33**	−.02					
6. Fear of closeness	2.34	.82	.**40**	.**17**	.**22**	.**45**	.08				
7. Pos psychof dissociation*	11.05	5.50	.**49**	.**23**	.**43**	.**15**	.**22**	.**25**			
8. Neg psychof dissociation*	12.59	6.84	.**49**	.**20**	.**37**	.**14**	.**19**	.**22**	.**82**		
9. Pos somatof dissociation*	2.94	.39	.**29**	.**14**	.**14**	.**10**	.**10**	.05	.**38**	.**42**	
10. Neg somatof dissociation*	3.87	.68	.**28**	.**15**	.**18**	.08	.**14**	.08	.**42**	.**51**	.**60**

*p* < .05, significant correlations are presented in bold; CPTSD = complex posttraumatic stress disorder; pos = positive; neg = negative; psychof = psychoform; somatof = somatoform; *scores are square rooted

### 2.2. Direct relationship of childhood trauma and CPTSD/DESNOS symptoms

The direct path between childhood trauma severity and CPTSD/DESNOS symptoms (Figure 1 Panel 1a) was statistically significant (*b* = 1.02, bootstrapped 95% CI [.72, 1.31], *SE b* = .15, β = .33, *p* < .001). Model fits were adequate (CFI = 1.00, RMSEA = .02, 90% CI [< .001, .08], SRMR = .02, AIC = 11,437.4, BIC = 11,470.3).

### 2.3. Unadjusted mediation model

When under-regulation, fear of abandonment and closeness, and positive and negative somatoform and psychoform dissociation were added to and statistically insignificant associations were removed from the model (*p* > .10), the direct path between childhood trauma and CPTSD/DESNOS symptoms remained statistically significant (*b* = .34, bootstrapped 95% CI [.10, .58], *SE b* = .12, β = .11, *p* = .005) (Figure 1 Panel 1a). An indirect path through under-regulation of affect was significant (*b* = .23, bootstrapped 95% CI [.13, .34], *SE b* = .05, β = .08, *p* < .001). An indirect relationship via fear of abandonment emerged (*b* = .14, bootstrapped 95% CI [.05, .23], *SE b* = .05, β = .05, *p* = .003). A third indirect path via fear of closeness was statistically significant (*b* = .10, bootstrapped 95% CI [.03, .17], *SE b* = .04, β = .03, *p* = .004), as was a fourth indirect path via negative psychoform dissociation (*b* = .15, bootstrapped 95% CI [.06, .24], *SE b* = .05, β = .05, *p* = .002). An indirect path via positive somatoform dissociation did not reach the conventional significance level but could be considered a trend (*b* = .04, bootstrapped 95% CI [-.01, .09], *SE b* = .02, β = .01, *p* = .08). This model showed adequate model fits (CFI = .96, RMSEA = .06, 90% CI [.04, .08], SRMR = .04, AIC = 18,701.7, BIC = 18,857.8). Next to depicting the mediation model for the relationship between childhood trauma and adult symptoms of CPTSD/DESNOS via affect dysregulation, relational fears and dissociation, Panel 1a also depicts all direct path estimates as standardized regression weights.

### 2.4. Mediation model adjusted for BPD symptom severity

When BPD symptom severity was added to the mediation model (Figure 1 Panel 1b), the direct path between childhood trauma and presence of CPTSD was still statistically significant (*b* = 1.02, bootstrapped 95% CI [.72, 1.31], SE *b* = .15, β = .33, *p* < .001; AIC = 15,671.7, BIC = 15,725.1). The direct path between childhood trauma severity and BPD symptoms was significant (*b* = .98, bootstrapped 95% CI [.76, 1.21], SE *b* = .11, β = .39, *p* < .001). When under-regulation of affect, fear of abandonment and closeness, and all features of dissociation were entered into the model and statistically insignificant associations were removed (*p*s > .10), the direct path between childhood trauma and CPTSD/DESNOS became smaller, but remained significant (*b* = .34, bootstrapped 95% CI [.10, .58], SE *b* = .12, β = .11, *p* = .005), as was the direct path to BPD symptoms (*b* = .49, bootstrapped 95% CI [.30, .68], SE *b* = .10, β = .20, *p* < .001).

As displayed in Figure 1 Panel 1b, four indirect mediation paths were identified; indirect paths emerged through under-regulation of affect (*b* = .24, bootstrapped 95% CI [.14, .34], SE *b* = .05, β = .08, *p* < .001), fear of abandonment (*b* = .10, bootstrapped 95% CI [.03, .17], SE *b* = .04, β = .03, *p* = .007), fear of closeness (*b* = .11, bootstrapped 95% CI [.03, .20], SE *b* = .05, β = .04, *p* < .001), and negative psychoform dissociation (*b* = .15, bootstrapped 95% CI [.06, .24], SE *b* = .05, β = .05, *p* = .002). The path via positive somatoform dissociation again approached but did not reach the conventional significance level (*b* = .05, bootstrapped 95% CI [.00, .09], SE *b* = .02, β = .08, *p* = .06). Model fits were adequate (CFI = 1.00, RMSEA = .02, 90% CI [< .001, .05], SRMR = .03, AIC = 25,595.9, BIC = 25,875.1). Next to depicting the mediation model adjusted for BPD symptoms, Panel 1b depicts all direct path estimates as standardized regression weights. Mediators of relationships between childhood trauma and CPTSD/DESNOS are shown in **bold black** font. Mediators of relationships between childhood trauma and BPD are in grey font. Shared mediators are in *italic*.

When the final model was specified with path estimates fixed at the values derived from the unadjusted mediation model, the resulting constrained mediation model is depicted in Figure 1 Panel 1c. A chi-squared test showed that the indirect paths did not differ (χ^2^ (20) = 13.28, *p* = .87) between the unconstrained (Panel 1b) and constrained (Panel 1c) models. Next to depicting the mediation model adjusted for BPD symptoms with path estimates constrained at values of the unadjusted model, Panel 1c shows all direct path estimates as standardized regression weights. Mediators of relationships between childhood trauma and CPTSD/DESNOS are shown in **bold black** font. Mediators of relationships between childhood trauma and BPD are in grey font. Shared mediators are in *italic.*

Thus, accounting for BPD-symptoms in the model did not change the mediation pathways between childhood trauma and CPTSD/DESNOS-symptoms.

## 3. Discussion

Study results demonstrate that three trans-diagnostic domains of functioning (i.e. affect regulation, dissociation, and relational impairment) that are associated with the DESNOS formulation of CPTSD and BPD statistically mediate the relationship between childhood trauma and CPTSD/DESNOS independent of the effects of BPD-symptoms. Although mediator variables linking childhood trauma to BPD and CPTSD/DESNOS were similar in several respects, they appear to represent distinct paths that are not simply a function of BPD. This inference is in line with previous studies that found that CPTSD/DESNOS can be differentiated from BPD (Cloitre et al., 2014; Van Dijke et al., 2012).

Evidence is mounting that the DESNOS formulation of CPTSD is not just a complicated array of overlapping psychological and personality symptoms, but a separate construct that is highly associated with prolonged childhood interpersonal trauma and chronic affect dysregulation, dissociation, and relational/attachment impairment (Ford & Courtois, 2014). Cloitre and colleagues (2014) identified four BPD-symptoms that distinguished an empirically-derived BPD sub-group from an empirically-derived CPTSD/DESNOS sub-group in a sample of women with childhood abuse histories: frantic efforts to avoid abandonment, unstable sense of self, unstable and intense interpersonal relationships, and impulsiveness. The present study’s findings are consistent with those of Cloitre et al. (2014) in that the DESNOS formulation of CPTSD involved problems with dissociation and emotion dysregulation that were distinct from BPD, but diverged from Cloitre et al. (2014) in demonstrating a potential link independent of BPD between fear of abandonment and relational impairment in the relationship between childhood trauma and the DESNOS formulation of CPTSD.

Hypothesis 1, that both overregulation and under-regulation of affect would be mediators, was only partially confirmed. Mediation was found for under-regulation of affect only, not for overregulation. Although the DESNOS formulation of CPTSD includes features of emotional down-regulation that are consistent with overregulation of affect, it appears that only the excitatory form of affect dysregulation (under-regulation) links childhood trauma to the DESNOS formulation of CPTSD, not the inhibitory form of overregulation. In earlier studies (Van Dijke, Ford, Frank, Van Son, & Van der Hart, 2013), overregulation was associated with BPD symptom severity but not with childhood trauma involving a primary caregiver. Under-regulation partially mediated the relationship between childhood trauma involving a primary caregiver and BPD-symptom severity. Thus, under-regulation of excitatory negative affect states among adults with childhood trauma histories may play a role in BPD, but under-regulation also appears to be a link between childhood trauma and the DESNOS formulation of CPTSD independent of BPD. Therefore, assessment (Ford, 2017a) and therapeutic interventions (Ford, 2017b) designed to address under-regulation of excitatory negative affect states and CPTSD/DESNOS appear warranted with adults in psychiatric treatment who have childhood trauma histories.

Hypothesis 2, that positive and negative somatoform and psychoform dissociation would mediate the relationship between childhood trauma and the DESNOS formulation of CPTSD, also was only partially confirmed. Somatoform dissociation was associated with CPTSD/DESNOS symptoms on a bivariate basis and approached statistical significance as a mediator, consistent with the somatic dysregulation features of the DESNOS formulation of CPTSD. However, these extreme forms of loss of somatic function and awareness were only weakly related to childhood trauma, which is consistent with prior findings of a limited relationship between childhood maltreatment and somatoform dissociation (Bohn, Bernardy, Wolfe, & Häuser, 2013; Van Dijke et al., 2015). These findings also are in line with the inconsistent relationship between psychoform dissociation and CPTSD/DESNOS symptoms reported in other investigations (e.g. Scoboria, Ford, Lin, & Frisman, 2008).

The current results also clarify prior findings showing that psychoform dissociation mediated the relationship between complex childhood trauma and CPTSD/DESNOS-symptoms (Van Dijke et al., 2015), demonstrating that it was negative psychoform dissociation (e.g. amnesia) and not positive psychoform dissociation (e.g. flashbacks) that mediated the relationship between childhood trauma and CPTSD/DESNOS when the effects of BPD symptoms were controlled. This is consistent with the inclusion of transient severe states of dissociation as a symptom of BPD, and suggests that the DESNOS formulation of CPTSD may share that type of positive psychoform dissociation with BPD but can be distinguished from BPD by inhibitory forms of psychoform dissociation that are more consistent with depersonalization or derealization. This further suggests that research is needed to clarify the relationship of the DESNOS formulation of CPTSD with the DSM-5 dissociative sub-type of PTSD (Lanius et al., 2010), particularly to determine if there are forms of negative psychoform dissociation that are shared or distinct in the two syndromes.

Hypothesis 3, that fear of closeness would be a mediator for the DESNOS formulation of CPTSD, was confirmed. However, fear of abandonment also consistently independently mediated the relationship between childhood trauma and CPTSD/DESNOS symptoms. Although BPD has been hypothesized to be differentiated from the DESNOS formulation of CPTSD based on intense fears of abandonment and rejection (Cloitre et al., 2014; Ford & Courtois, 2014), fear of abandonment also appears to provide a link between childhood trauma and CPTSD/DESNOS. Nevertheless, this link was independent of the severity of BPD symptoms, suggesting that fears of abandonment (and of closeness) provide a connection between childhood trauma and CPTSD/DESNOS that is not accounted for by the parallel mediation pathways between childhood trauma and BPD through these fears. Research is needed to determine how, and for whom, adult relational fears related to childhood trauma contribute to the DESNOS formulation of CPTSD versus BPD (or to both). Both fear of abandonment and fear of closeness warrant further research as adult sequelae of childhood trauma and specifically in relation to the DESNOS formulation of CPTSD.

Study findings should be interpreted in light of several limitations. These include the use of a self-report version of the SIDES, rather than the interviewer version validated by Ford and Kidd (1998), reliance on clinician rather than research ratings of the diagnostic variables, use of retrospective self-report to assess trauma history, and inclusion of only two specific psychiatric diagnoses and the general class of severe affective/anxiety disorders within the study sample. Prospective research documenting the longitudinal relationships of childhood trauma, mechanisms of self-regulation, and both CPTSD/DESNOS and BPD are needed in order to confirm mediated relationships suggested by the cross-sectional results from this study. However, the study’s strengths include a large sample with precisely documented index diagnoses, detailed (and reliable, when collateral confirmation was possible) assessment of childhood trauma history, and use of the SIDES to assess all CPTSD features.

Clinically, these findings suggest that under-regulation of affect and adult relational fears warrant careful assessment and targeted treatment for adults with the DESNOS formulation of CPTSD independent of the severity of their BPD symptoms (Ford & Courtois, 2014). The socio-psychobiological adaptations that ensue when interpersonal trauma occurs during formative or sensitive developmental periods involve an increased preparedness for threat that includes a heightened salience and intensity of negative affects (Langevin et al., 2015; Shields & Cicchetti, 1998) and anticipation of relational betrayal (Freyd, DePrince, & Gleaves, 2007). These defensive (self-protective) adaptations to childhood trauma may fundamentally alter an individual’s ways of coping with stress without resulting in characterological problems that would warrant a BPD diagnosis (Ford & Courtois, 2014). Thus, the DESNOS formulation of CPTSD provides a basis for identifying and treating chronic trauma-related affect and interpersonal dysregulation in adulthood without presuming that these are the signs or results of personality disorder (Ford, 2015, 2017a).

Therapies designed to address negative psychoform dissociation (e.g. present-focused approaches to enhancing narrative autobiographical memory and integration of fragmented or impaired conscious awareness of self and life circumstances; Ford, 2017b) also may be warranted important for adults with the DESNOS formulation of CPTSD in order to enhance treatment effectiveness and prevent early termination of therapy. Trauma memory processing therapies are designed to reduce positive psychoform symptoms of PTSD (e.g. intrusive flashbacks) but may be difficult if negative psychoform symptoms, such as amnesia or derealization, undermine memory processing capacities. Although the link between childhood trauma and positive symptoms of somatoform dissociation (e.g. medically unexplained pain) was tenuous in the current study, there is evidence that childhood trauma is associated with physical as well as emotional pain (Nelson, Cunningham, & Kashikar-Zuck, 2017; Sachs-Ericsson, Sheffler, Stanley, Piazza, & Preacher, 2017) and that perceived control over pain is associated with reduced anxiety and enhanced emotion regulation (Salomons, Nusslock, Detloff, Johnstone, & Davidson, 2015). Therefore, assessment of somatoform dissociation appears warranted as a precaution when clinically treating adults with CPTSD/DESNOS.

### 3.1. Conclusion

**Figure 2.**
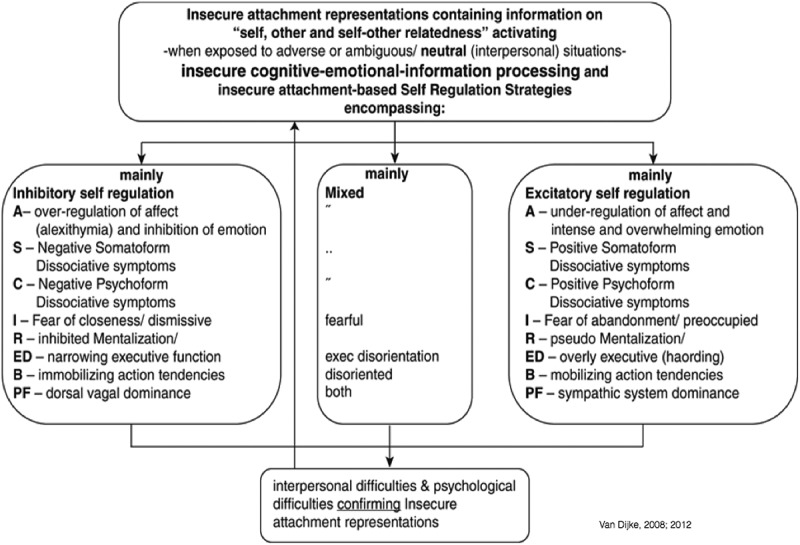
Dysfunctional regulation operating in vicious cycles. A = affect and emotion dysregulation; S = somatic/bodily symptoms; C = cognitive symptoms; I = relational impairment; R = reflective difficulties; ED = executive dysfunction; B = behavioural action tendencies; PF = psycho-physiological symptoms. Van Dijke (2008) described dysfunctional regulation as operating in vicious cycles that approach the long-term sequelae of trauma-by-primary-caretaker from a developmental perspective. Dysfunctional regulation may present in patients in three qualitatively different forms: Inhibitory, Excitatory, and combined Inhibitory & Excitatory (IE) regulation. Symptoms include disturbances in self-regulation across several domains of functioning including affective, cognitive, somatic, relational, reflective, executive, behavioural, and psycho-physiological functioning. Activation of dysfunctional regulation seems to follow trauma-by-primary-caretaker associated negatively-biased cognitive-emotional information processing. However, when potentially neutral situations are processed and evaluated as threatening or potentially harmful, dysfunctional regulation is activated false positively. Inhibitory regulation when activated based upon biased (negative avoidant) cognitive-emotional information processing encompasses, among others, overregulation of affect, negative psychoform and somatoform dissociation, fear of closeness in adult relationships, inhibited mentalization, narrowed executive functioning, immobilizing action tendencies, and dominance of the sympathetic system. Consequently, this results in interpersonal misunderstanding and disappointments, which in turn condition and uphold the insecure attachment representation/working models turning into inhibitory regulation vicious circle. Excitatory regulation when activated based upon biased (negative-anxious) cognitive-emotional information processing encompasses e.g. under regulation of affect, positive psychoform and somatoform dissociation, fear of abandonment in adult relationships, pseudo mentalization, overly executive functioning, mobilizing action tendencies, and dominance of the dorsal vagal system. Consequently, this results in interpersonal misunderstanding and disappointments, which in turn conditions and upholds the insecure attachment representation/working models turning into an excitatory regulation vicious circle. Combined Inhibitory & Excitatory (IE) regulation encompasses both inhibitory and excitatory domains and symptoms that can present alternating or in combinations in patients. It should be noted that dysfunctionally-regulated persons, when confronted with internal or external adverse events, risk to never meet the sense of personal efficacy, resilience, and optimism. Figure 2 summarizes the hypothesized relationships for dysfunctional regulations discussed above.Assessing the relations between childhood trauma and adult complex CPTSD direct and indirect through mediators affect dysregulation, dissociation, and attachment fearsAssessing these direct and indirect relations while correcting for BPD symptomsAssessing similarities and differences for the direct and indirect relations between childhood trauma and CPTSD and/or BPD

Relationships between childhood exposure to interpersonal trauma and CPTSD/DESNOS symptoms in adulthood through three mediators i.e. relational impairment, affect dysregulation, and identity alterations – in their separate inhibitory and excitatory forms (Figure 2) *–* were found, independent of the effects of BPD-symptoms.
